# C/EBPβ Promotes Transition from Proliferation to Hypertrophic Differentiation of Chondrocytes through Transactivation of p57^Kip2^

**DOI:** 10.1371/journal.pone.0004543

**Published:** 2009-02-20

**Authors:** Makoto Hirata, Fumitaka Kugimiya, Atsushi Fukai, Shinsuke Ohba, Naohiro Kawamura, Toru Ogasawara, Yosuke Kawasaki, Taku Saito, Fumiko Yano, Toshiyuki Ikeda, Kozo Nakamura, Ung-il Chung, Hiroshi Kawaguchi

**Affiliations:** 1 Departments of Sensory & Motor System Medicine, Faculty of Medicine, University of Tokyo, Bunkyo-ku, Tokyo, Japan; 2 Center for Disease Biology and Integrative Medicine, Faculty of Medicine, University of Tokyo, Bunkyo-ku, Tokyo, Japan; Baylor College of Medicine, United States of America

## Abstract

**Background:**

Although transition from proliferation to hypertrophic differentiation of chondrocytes is a crucial step for endochondral ossification in physiological skeletal growth and pathological disorders like osteoarthritis, the underlying mechanism remains an enigma. This study investigated the role of the transcription factor CCAAT/enhancer-binding protein β (C/EBPβ) in chondrocytes during endochondral ossification.

**Methodology/Principal Findings:**

Mouse embryos with homozygous deficiency in C/EBPβ (C/EBPβ−/−) exhibited dwarfism with elongated proliferative zone and delayed chondrocyte hypertrophy in the growth plate cartilage. In the cultures of primary C/EBPβ−/− chondrocytes, cell proliferation was enhanced while hypertrophic differentiation was suppressed. Contrarily, retroviral overexpression of C/EBPβ in chondrocytes suppressed the proliferation and enhanced the hypertrophy, suggesting the cell cycle arrest by C/EBPβ. In fact, a DNA cell cycle histogram revealed that the C/EBPβ overexpression caused accumulation of cells in the G0/G1 fraction. Among cell cycle factors, microarray and real-time RT-PCR analyses have identified the cyclin-dependent kinase inhibitor p57^Kip2^ as the transcriptional target of C/EBPβ. p57^Kip2^ was co-localized with C/EBPβ in late proliferative and pre-hypertrophic chondrocytes of the mouse growth plate, which was decreased by the C/EBPβ deficiency. Luciferase-reporter and electrophoretic mobility shift assays identified the core responsive element of C/EBPβ in the p57^Kip2^ promoter between −150 and −130 bp region containing a putative C/EBP motif. The knockdown of p57^Kip2^ by the siRNA inhibited the C/EBPβ-induced chondrocyte hypertrophy. Finally, when we created the experimental osteoarthritis model by inducing instability in the knee joints of adult mice of wild-type and C/EBPβ+/− littermates, the C/EBPβ insufficiency caused resistance to joint cartilage destruction.

**Conclusions/Significance:**

C/EBPβ transactivates p57^Kip2^ to promote transition from proliferation to hypertrophic differentiation of chondrocytes during endochondral ossification, suggesting that the C/EBPβ-p57^Kip2^ signal would be a therapeutic target of skeletal disorders like growth retardation and osteoarthritis.

## Introduction

Most skeletal growth is achieved by endochondral ossification. During the process, chondrocytes undergo proliferation, hypertrophic differentiation, and apoptosis [1], each of which is regulated by distinct signals. Among them, chondrocyte hypertrophy is a rate-limiting step for the skeletal growth, being responsible for 40–60% of the endochondral ossification [2], [3]. The initiation is precisely linked with the cessation of proliferation; however, the molecular mechanism underlying the harmonious transition from the proliferation to hypertrophic differentiation of chondrocytes remains an enigma.

CCAAT/enhancer-binding protein β (C/EBPβ), also known as nuclear factor-interleukin-6 (NF-IL6), is a member of the C/EBP family of six transcription factors characterized by a carboxyl-terminal leucine zipper dimerization domain and an adjacent highly conserved basic DNA binding domain [4]–[6]. Contrary to C/EBPα that is purely antiproliferative as a tumor suppressor in several cell types, C/EBPβ regulates expression of various genes involved in cell differentiation, proliferation, survival, immune function and female reproduction, as well as tumor invasiveness and progression, through a variety of mechanisms [6].Over the past several years, C/EBPβ has been shown to control differentiation of hematopoietic and adipogenic cells [7], [8]. The present study initially investigated skeletal phenotype of C/EBPβ-deficient (C/EBPβ−/−) mice which have been reported to display mainly hematopoietic and adipogenic defects [9]–[11]. The mice showed dwarfism with an elongated proliferative zone and delayed chondrocyte hypertrophy in the limb cartilage, implicating the cell cycle control by C/EBPβ in chondrocytes.

Cell cycle factors appear to play an important role in the control of chondrocyte proliferation and differentiation [12], [13]. During the cell cycle activation, complexes of cyclin and cyclin-dependent kinase (CDK) promote G1/S-phase transition from G0/G1 by phosphorylating Rb-related pocket proteins, which activate genes required for the S-phase entry. The cyclin-CDK complexes are inhibited by two major families of CDK inhibitors [14]. The p16 INK4 family specifically binds and inactivates monomeric CDK4 or CDK6, whereas the Cip/Kip family, which includes p21^Cip1^, p27^Kip1^, and p57^Kip2^ (p57), inhibits all G1/S-phase cyclin-CDK complexes. Since the control of these cell cycle factors driving S-phase onset greatly influences the commitment to cell differentiation, the present study performed a screen of potential transcriptional targets of C/EBPβ using a microarray analysis, and identified p57 as the most probable target during hypertrophic differentiation of chondrocytes. We further investigated the molecular mechanism underlying the regulation of skeletal growth and endochondral ossification through the C/EBPβ-p57 signal in chondrocytes.

## Results

### C/EBPβ−/− mice exhibit impaired skeletal growth and endochondral ossification

**Figure 1:**
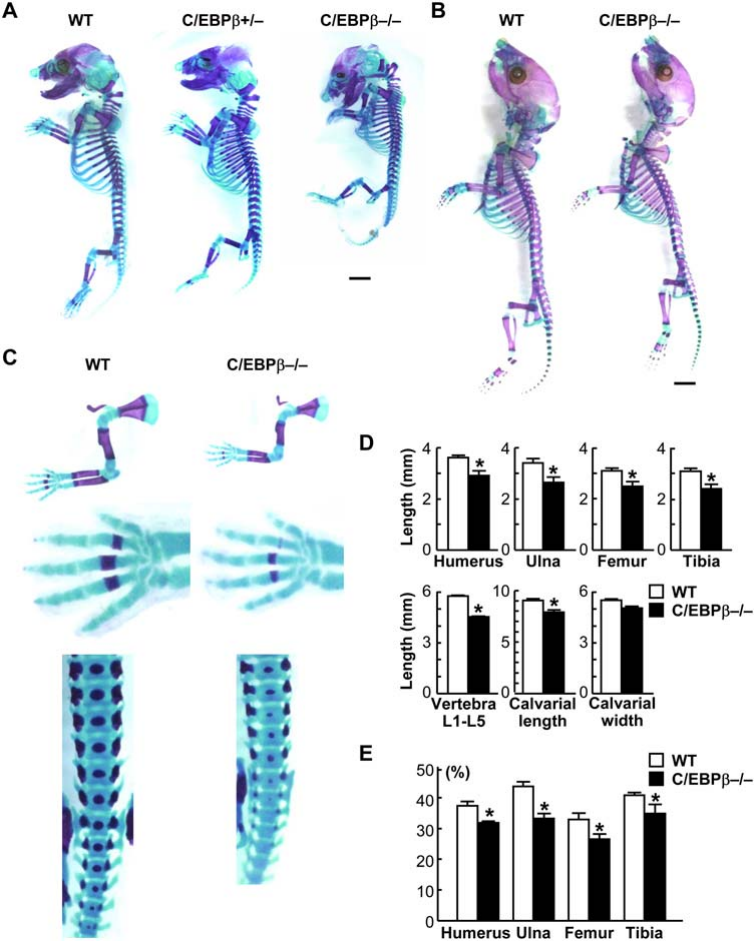
C/EBPβ−/− mice exhibit impaired skeletal growth and endochondral ossification. (A, B) Double stainings with Alizarin red and Alcian blue of the whole skeleton of the wild-type (WT), C/EBPβ+/−, and C/EBPβ−/− littermates at E16.5 (A) and at 3 d after birth (B). Scale bar, 2 mm. (C) Double stainings of the upper limbs, hands, and lumbar spines of the two genotypes. (D) Length of humerus, ulna, femur, tibia, vertebra (1st to 5th lumbar spines), and the calvarial length and width of the WT and C/EBPβ−/− littermates. (E) The percent ratio of Alizarin red-positive mineralized area to total length of the long bones of the two genotypes. Data are expressed as means (bars)±SEM (error bars) of 4 bones per genotype. *P<0.01 vs. WT.

To analyze the physiological role of C/EBPβ in skeletal growth and endochondral ossification, we investigated the skeletal phenotypes of heterozygous and homozygous C/EBPβ-deficient (C/EBPβ+/− and C/EBPβ−/−) mice. Although the C/EBPβ+/− skeleton was normal, C/EBPβ−/− mice exhibited dwarfism as compared to the wild-type littermates from embryonic stages (Figure 1A). After birth, however, the skeletal size of C/EBPβ−/− mice gradually caught up with that of the wild type littermates (Figure 1B), and they became similar after 1 week of age. At the embryos, the limbs and vertebrae which are known to be primarily formed through endochondral ossification were about 20–25% shorter in C/EBPβ−/− mice than the wild-type, although calvarial growth, especially the width, formed through endochondral ossification and intramembranous ossification did not show such a difference (Figure 1D). Skeletal double staining revealed that not only the total bone length, but also the ratio of mineralized area shown by the positive Alizarin red staining to the total length was decreased, confirming that endochondral ossification was impaired by the C/EBPβ deficiency (Figure 1C, E).

### Hypertrophic differentiation of chondrocytes is delayed in the C/EBPβ−/− limb cartilage

**Figure 2:**
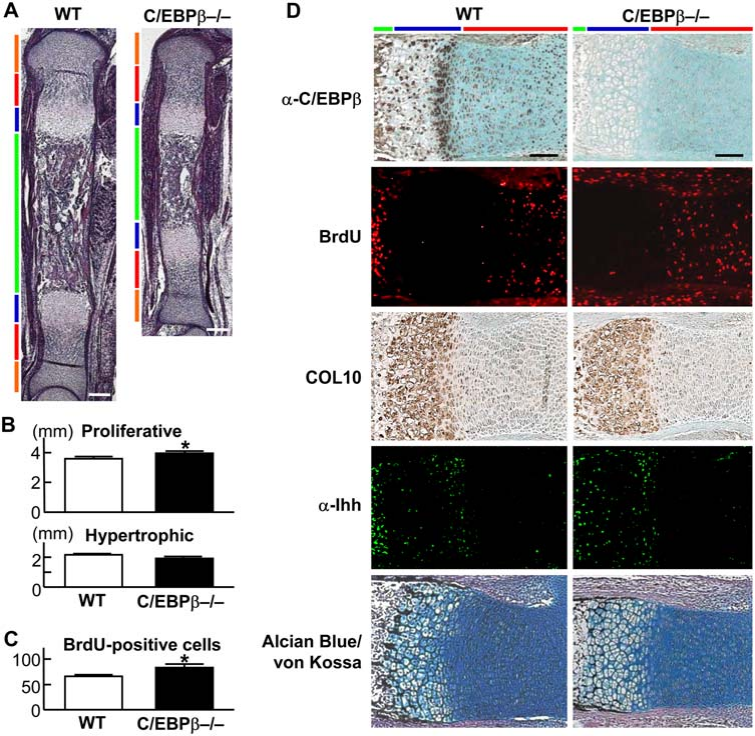
Hypertrophic differentiation of chondrocytes is delayed in the C/EBPβ−/− limb cartilage. (A) HE staining of whole tibias of wild-type (WT) and C/EBPβ−/− littermate embryos (E16.5). Orange, red, blue, and green bars indicate layers of resting zone, proliferative zone, hypertrophic zone, and bone area, respectively. Scale bars, 200 µm. (B) Length of proliferative and hypertrophic zones of the two genotypes. (C) Number of BrdU-positive cells in the proximal tibia of the two genotypes. Data are expressed as means (bars)±SEM (error bars) of 5 mice per genotype. *P<0.05 vs. WT. (D) Immunostaining with an antibody to C/EBPβ (α-C/EBPβ), BrdU labeling, in situ hybridization of type X collagen (COL10), immunostaining with an antibody to Ihh (α-Ihh), and Alcian blue/von Kossa double stainings of the tibial cartilage in two genotypes. Color bars indicate layers as above. Scale bars, 100 µm.

To know the mechanism underlying the impaired skeletal growth in C/EBPβ−/− mice, we compared the tibial limb cartilage of the wild-type and C/EBPβ−/− littermates at E16.5 (Figure 2A). Among the resting, proliferative, hypertrophic zones, and bone area, the proliferative zone was elongated while the hypertrophic zone was normal in the C/EBPβ−/− limb (Figure 2B). The number of proliferating chondrocytes with BrdU uptake was actually increased in the C/EBPβ−/− cartilage (Figure 2C).

C/EBPβ was shown by immunohistochemistry to be localized predominantly in late proliferative and pre-hypertrophic chondrocytes of the wild-type cartilage, but not in the C/EBPβ−/− cartilage (Figure 2D, top). Further histological examination by BrdU labeling, in situ hybridization of type X collagen (COL10), immunohistochemistry of indian hedgehog (Ihh), and Alcian blue/von Kossa double stainings supported the elongation of the proliferative zone and delay of chondrocyte hypertrophy by the C/EBPβ deficiency (Figure 2D).

### C/EBPβ inhibits proliferation and promotes hypertrophic differentiation in cultured primary chondrocytes

**Figure 3:**
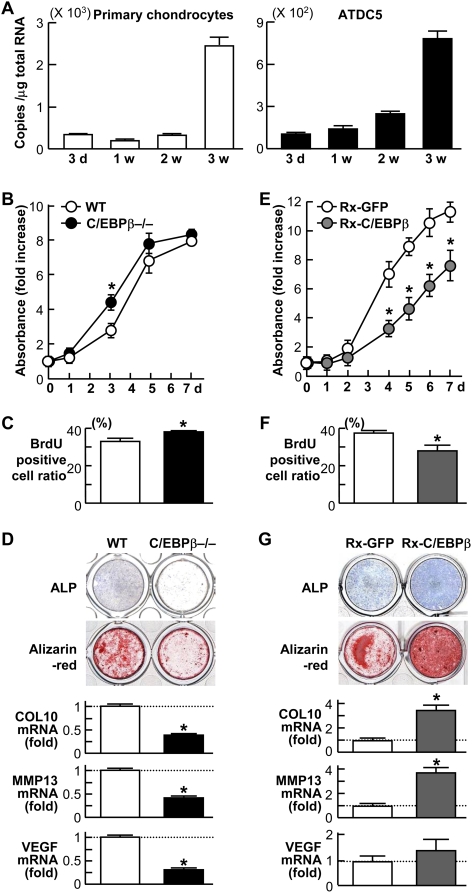
C/EBPβ inhibits proliferation and promotes hypertrophic differentiation in cultured primary chondrocytes. (A) Time course of C/EBPβ mRNA level determined by real-time RT-PCR analysis during differentiation of primary chondrocytes and ATDC5 cells cultured for 3 weeks with insulin. (B) Growth curves by the XTT assay of primary chondrocytes derived from ribs of wild-type (WT) and C/EBPβ−/− littermates. (C) Ratio of BrdU-positive cells to total cells after 3 d culture of primary chondrocytes derived from WT and C/EBPβ−/− ribs. (D) ALP and Alizarin red stainings, and relative mRNA levels of COL10, MMP13, and VEGF of the primary chondrocytes from the two genotypes determined by real-time RT-PCR analysis at 2 weeks of culture after confluency. (E) Growth curves of primary WT rib chondrocytes with retroviral transfection of C/EBPβ (Rx-C/EBPβ) or the control GFP (Rx-GFP). (F) Ratio of BrdU-positive cells to total cells after 4 d culture of primary WT rib chondrocytes with Rx-C/EBPβ or Rx-GFP. (G) ALP and Alizarin red stainings, and relative mRNA levels of the chondrocyte hypertrophy markers of the rib chondrocytes with Rx-C/EBPβ or Rx-GFP at 2 weeks of culture after confluency. All data are expressed as means (symbols or bars)±SEM (error bars) of 6 wells or dishes per group. *P<0.01 vs. WT or Rx-GFP.

When primary chondrocytes derived from mouse ribs and mouse chondrogenic cell line ATDC5 were cultured in the differentiation medium, the C/EBPβ mRNA level was increased with the differentiation (Figure 3A), which was comparable to the in vivo expression pattern of the limb cartilage.

We then examined the effects of loss- and gain-of-functions of C/EBPβ on proliferation and hypertrophic differentiation of the primary rib chondrocytes. When chondrocytes from wild-type and C/EBPβ−/− littermates were compared, cell number determined by the XTT assay was enhanced in the C/EBPβ−/− chondrocytes at 3 d of culture (Figure 3B). The percentage of BrdU-positive cells was also increased in the C/EBPβ−/− culture at this time point (Figure 3C), indicating that the increased cell number was due to the enhanced proliferation, rather than the effect on cell survival, vitality, or apoptosis. Contrarily, hypertrophic differentiation determined by alkaline phosphatase (ALP) and Alizarin red stainings, and mRNA levels of COL10, matrix metalloproteinease-13 (MMP13) and vascular endothelial growth factor (VEGF), parameters of chondrocyte hypertrophy, were suppressed by the deficiency (Figure 3D).

In contrast, retroviral overexpression of C/EBPβ in the wild-type rib chondrocytes suppressed the proliferation and enhanced the hypertrophic differentiation parameters (Figure 3E, F, and G). Collectively, C/EBPβ was shown to be essential for cessation of proliferation and promotion of hypertrophic differentiation, suggesting arrest of the cell cycle and exit from it.

### C/EBPβ regulates cell cycle and p57 as the transcriptional target

**Figure 4:**
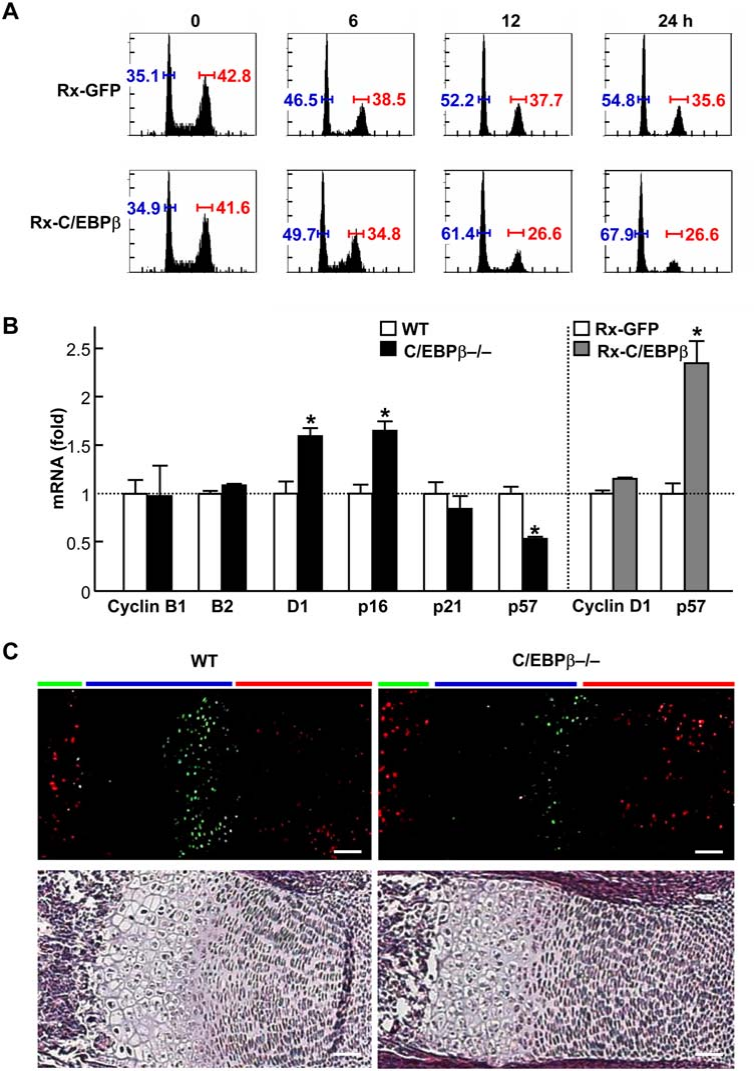
C/EBPβ affects cell cycle factors. (A) Time course of DNA histograms by a flow cytometric analysis of C3H10T1/2 cells with retroviral transfection of C/EBPβ (Rx-C/EBPβ) or the control GFP (Rx-GFP) after synchronization at the G2/M phase by nocodazole treatment. The horizontal and vertical axes represent the DNA content and relative frequency, respectively. The blue and red bars indicate the rates of cells in G0/G1 and G2/M phases, respectively. (B) Effects of loss- and gain-of-functions of C/EBPβ on relative mRNA levels of cell cycle factors that were identified as possible transcriptional targets of C/EBPβ by a microarray analysis (Table S1). The levels were compared by real-time RT-PCR analysis in the cultures between wild-type (WT) and C/EBPβ−/− rib chondrocytes (left), and between WT rib chondrocytes with Rx-C/EBPβ and Rx-GFP (right). Data are expressed as means (bars)±SEM (error bars) of 6 samples per group. (C) Double immunofluorescence of p57 (green) and BrdU (red) in the proximal cartilage of tibias of the two genotype embryos (E16.5) and the HE staining (bottom) as a reference. Red, blue, and green bars indicate layers of proliferative zone, hypertrophic zone, and bone area, respectively. Scale bars, 50 µm.

We therefore examined the regulation of cell cycle by C/EBPβ. A DNA cell cycle histogram in mouse mesenchymal C3H10T1/2 cells after the cycle synchronization revealed that the C/EBPβ overexpression enhanced accumulation of cells in the G0/G1 fraction (Figure 4A). To identify cell cycle factors lying downstream of the C/EBPβ signal, we performed a screen of transcriptional targets of C/EBPβ using a microarray analysis (Table S1). The C/EBPβ overexpression caused downregulation of cyclin B1, B2 and D1, and upregulation of the cyclin-dependent kinase inhibitors p16, p21 and p57, by 50% or more as compared to the empty vector overexpression. Since the above analyses were performed in non-chondrogenic C3H10T1/2 cells, we further examined the expressions of the candidate genes by real-time RT-PCR analysis in the cultures between wild-type and C/EBPβ−/− rib chondrocytes (Figure 4B). Cyclin B1, B2, and p21 were not significantly altered by the C/EBPβ deficiency, while p16 showed contradictory upregulation. Cyclin D1 and p57 were confirmed to be upregulated and downregulated, respectively, by the loss-of-function of C/EBPβ. However, when the expressions were further compared between primary chondrocytes with retroviral overexpression of C/EBPβ and the control GFP, the cyclin D1 was not downregulated, whereas p57 was upregulated by the C/EBPβ overexpression. These indicate that p57 was the only cell cycle factor whose expression was confirmed to be regulated positively and negatively by the gain- and loss-of-functions of C/EBPβ, respectively. Double immunofluorescence of p57 and BrdU in the wild-type cartilage revealed that p57 was localized predominantly in late proliferative and pre-hypertrophic chondrocytes which do not exhibit BrdU uptake (Figure 4C). The p57 expression was confirmed to be decreased in the C/EBPβ−/− cartilage.

### C/EBPβ transactivates p57 through direct binding to a C/EBP motif

**Figure 5:**
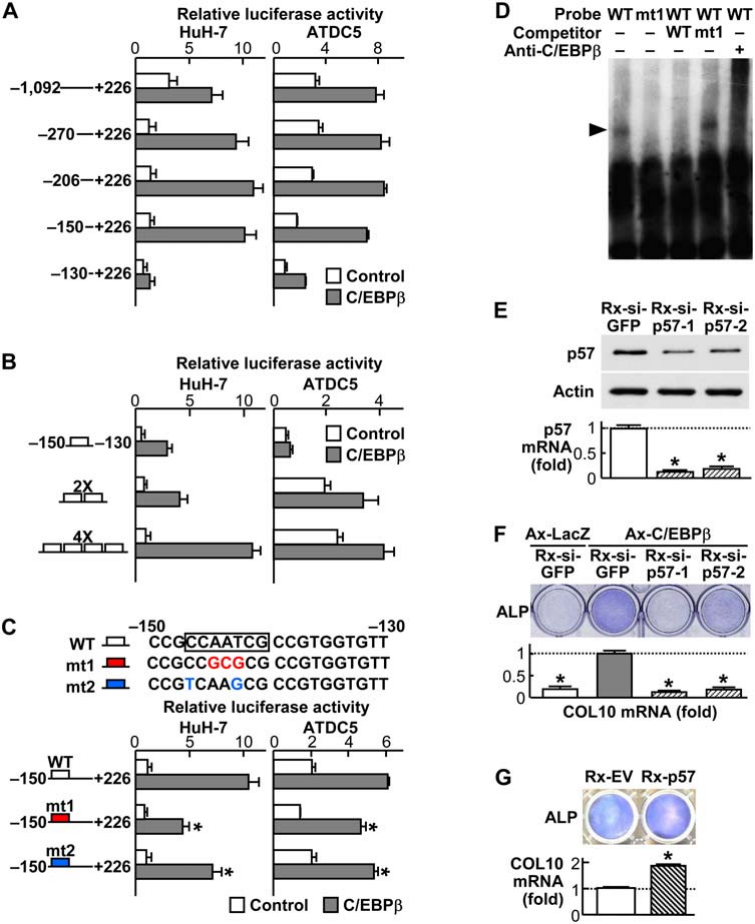
C/EBPβ transactivates p57 through binding to a C/EBP motif and the C/EBPβ-p57 signal induces chondrocyte hypertrophic differentiation. (A) Deletion analysis using luciferase-reporter constructs containing the 5′-flanking sequences from −1,092 to +226 bp of the p57 promoter and a series of deletion fragments in HuH-7 and ATDC5 cells transfected with C/EBPβ or the control GFP. (B) Dose-response analysis of the tandem repeats of the identified responsive element (−150/−130) by the luciferase-reporter assay in the HuH-7 and ATDC5 cells. (C) Site-directed mutagenesis analysis by two mutations (mt1 and mt2) in the C/EBPβ motif (−147/−141) as compared to the wild-type (WT) construct by the luciferase-reporter assay in HuH-7 and ATDC5 cells. *P<0.01 vs. WT-C/EBPβ. (D) EMSA for specific binding of the nuclear extract from C/EBPβ-transfected ATDC5 cells with the oligonucleotide probe (−160/−120) containing the WT or mt1 C/EBPβ motif. The arrowhead indicates the complex. Cold competition with 50-fold excess of unlabeled WT or mt1 probe, and supershift by an antibody to C/EBPβ of the complex are also presented. (E) The protein and mRNA levels of p57 determined by immunoblotting and real-time RT-PCR, respectively, by the p57 siRNA. Stable lines of C3H10T1/2 cells retrovirally transfected with two kinds of siRNA of p57 (Rx-si-p57-1 and Rx-si-p57-2) or the control GFP siRNA (Rx-si-GFP) were established. *P<0.01 vs. Rx-si-GFP. (F) Effects of the p57 siRNA above on the C/EBPβ-induced hypertrophic differentiation of chondrocytes. Primary rib chondrocytes were transfected with Rx-si-p57-1, Rx-si-p57-2, or Rx-si-GFP, and further adenovirally co-transfected with C/EBPβ or the control LacZ (Ax-C/EBPβ or Ax-LacZ). Hypertrophic differentiation was determined by ALP staining and relative COL10 mRNA level by real-time RT-PCR analysis at 2 weeks of culture after confluency. *P<0.01 vs. Ax-C/EBPβ with Rx-si-GFP. (G) Hypertrophic differentiation of ATDC5 cells stably transfected with the retrovirus expressing p57 (Rx-p57) or the empty vector (Rx-EV) cultured for 3 weeks with insulin and further for 2 d with inorganic phosphate. *P<0.01 vs. Rx-EV. All graphs are expressed as means (bars)±SEM (error bars) for 6 wells/group.

To know the mechanism underlying the induction of p57 expression by C/EBPβ, we analyzed the promoter activity of p57 using human hepatoma HuH-7 cells and ATDC5 cells transfected with a luciferase reporter gene construct containing the 5′-flanking sequences from −1,092 to +226 bp of the p57 promoter (Figure 5A). The transcriptional activity determined by the luciferase-reporter assay was enhanced by co-transfection with C/EBPβ in both cells, indicating the transcriptional induction of p57 by C/EBPβ. Deletion analysis by a series of 5′-deletion constructs identified the responsive element to C/EBPβ as being located between −150 and −130 bp region. The tandem-repeat constructs of this region were confirmed to respond to the C/EBPβ overexpression depending on the repeat number in both cells (Figure 5B). As this region contained a putative C/EBP-binding motif [15], the site-directed mutagenesis was carried out by creating two mutations in the motif. Both of the mutations caused partial but significant suppression of the promoter activity in both cells, indicating that the C/EBP motif is a responsive element (Figure 5C). EMSA revealed the specific binding of the nuclear extract from C/EBPβ-overexpressed ATDC5 cells with the oligonucleotide probe containing the identified responsive element above (Figure 5D). The mutagenesis in the C/EBP motif of the probe resulted in a failure to form the complex. Cold competition with excess amounts of an unlabeled wild-type probe, but not the mutated probe, suppressed the complex formation, confirming the specific binding to the C/EBPβ motif. Specificity of C/EBPβ binding was further verified by the antibody supershift. These lines of results demonstrate that C/EBPβ transactivates the p57 promoter, at least in part, through direct binding to a C/EBP motif between the −150 and −130 bp region.

### The C/EBPβ-p57 signal induces chondrocyte hypertrophic differentiation

To know the functional interaction between C/EBPβ and p57 during chondrocyte hypertrophic differentiation, we established two small interfering RNA (siRNA) constructs of p57 for the gene silencing. We initially confirmed significant decreases of p57 protein and mRNA levels by stable transfection of the two siRNAs (Figure 5E). The C/EBPβ-induced hypertrophic differentiation of cultured rib chondrocytes determined by ALP staining and COL10 expression was suppressed by the p57 knockdown through the siRNA (Figure 5F), indicating the mediation of p57 in the C/EBPβ induction of hypertrophic differentiation. We confirmed that retroviral overexpression of p57 enhanced the hypertrophy markers in cultured ATDC5 cells (Figure 5G).

### C/EBPβ is involved in cartilage destruction during osteoarthritis progression

**Figure 6:**
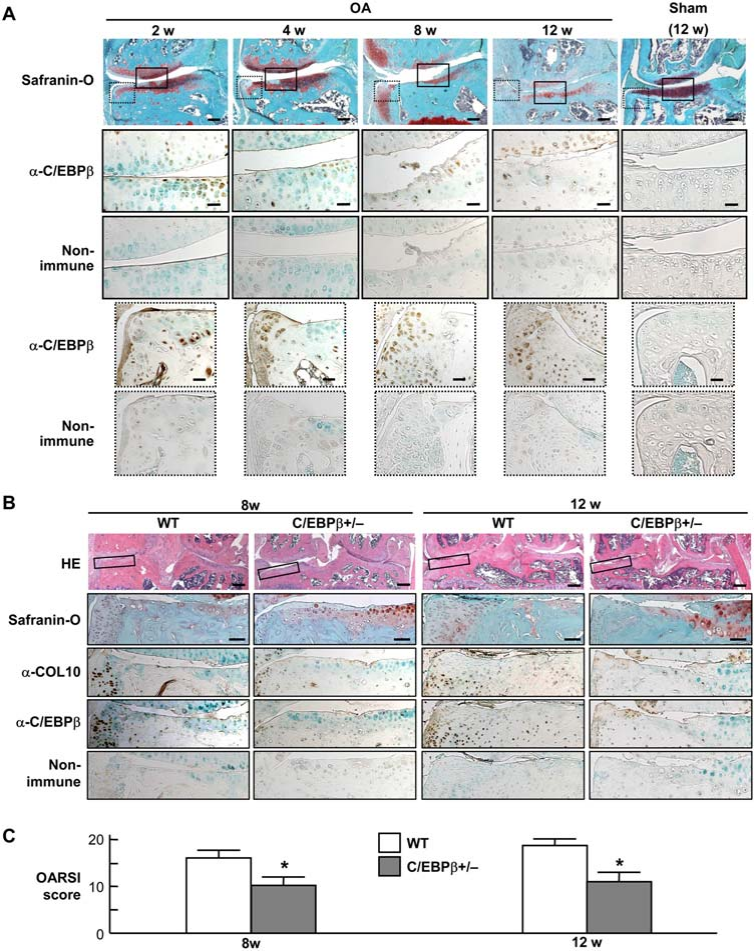
C/EBPβ is involved in cartilage destruction during osteoarthritis progression. (A) Time course of joint cartilage destruction and C/EBPβ expression in the medial portion of cartilage after creating an experimental osteoarthritis model that induces instability to the knee joints of 8-week-old wild-type mice. Safranin-O staining and immunohistochemical staining with an antibody to C/EBPβ (α-C/EBPβ) or the non-immune control IgG were performed at the indicated weeks after surgery. A sham operation was performed using the same approach, and assessed after 12 weeks. Boxed areas with solid and dotted lines in the top row indicate the regions of the other rows. Scale bars, 100 µm (top) and 400 µm (others). (B) HE and Safranin-O stainings, and immunohistochemical stainings with α-C/EBPβ and α-COL10 or the non-immune IgG in the tibial cartilage of wild-type (WT) and C/EBPβ+/− littermates 8 weeks and 12 weeks after the surgery. Boxed areas in the top row indicate the regions of the other rows. Scale bars, 200 µm (top), 400 µm (others). (C) Cartilage destruction according to the OARSI grading system. Data are expressed as means (bars)±SEM (error bars) for 5 mice per genotype at 8 and 12 weeks. *P<0.05 vs. WT

In addition to the physiological role in skeletal growth in embryos, we finally examined the contribution of C/EBPβ in chondrocytes under pathological conditions. We and others have reported that endochondral ossification including chondrocyte hypertrophy is a crucial step for cartilage destruction during osteoarthritis progression [16]–[20]. We therefore created an experimental osteoarthritis model that induces instability to the knee joints in 8-week-old wild-type mice [17], [21], and found that C/EBPβ was localized at the frontline of cartilage degradation in the central and peripheral areas of the joint cartilage during osteoarthritis progression (Figure 6A). To know the functional involvement of C/EBPβ under the pathological conditions, we compared the cartilage destruction between C/EBPβ+/− and the wild-type littermates that showed similar phenotypes under physiological conditions (Figure 1A) [9]. C/EBPβ−/− mice were not used in this experiment since their skeleton was originally small, the joint shape was abnormal, and the activity was low, so that mechanical stress caused by the joint instability was not assumed to be comparable to that of wild-type mice. The cartilage destruction as well as COL10 expression was suppressed in C/EBPβ+/− mice, remaining a substantial undegraded matrix even 8 to 12 weeks after the surgery (Figure 6B). Quantification using the OARSI grading system [22] confirmed significant prevention of cartilage destruction by the C/EBPβ haploinsufficiency (Figure 6C).

## Discussion

The present study for the first time demonstrated that the transcription factor C/EBPβ is essential for physiological skeletal growth and endochondral ossification by analyses of the deficient mice. This function was dependent on the promotion of transition from proliferation to hypertrophic differentiation of chondrocytes through the cell cycle control. Our further screening of cell cycle factors identified the cyclin-dependent kinase inhibitor p57 as the transcriptional target, and detected a responsive element of C/EBPβ in the promoter. We finally showed the functional mediation of p57 in the C/EBPβ action, and confirmed the importance of the C/EBPβ-p57 signal in the chondrocyte hypertrophy during skeletal growth and osteoarthritis progression.

Growth retardation of C/EBPβ−/− mice was seen during embryogenesis only and disappeared as the animals grew up after birth under physiological conditions (Figure 1A & B). This may possibly be due to compensatory mechanisms by other C/EBP family members which are known to control cellular differentiation in several lineages [23]–[26]. Regarding the mesenchymal cell lineage, C/EBPδ has been reported to show similar and compensatory actions for adipogenic and osteogenic differentiation [11], [27]–[31]. Since the involvement of C/EBPδ in chondrogenic differentiation from the mesenchymal precursors remains unknown, we initially examined the expression by immunohistochemistry in the limb cartilage (E16.5) (Figure S1A). It was expressed predominantly in late proliferative and pre-hypertrophic chondrocytes, similarly to the C/EBPβ expression, and this was not altered in the C/EBPβ−/− cartilage. In addition, retroviral overexpression of C/EBPδ enhanced hypertrophic differentiation determined by COL10 and MMP13 mRNA levels in cultured ATDC5 cells (Figure S1B). Furthermore, the p57 promoter activity was enhanced by the C/EBPδ overexpression, although the effect was somewhat weaker than that by C/EBPβ (Figure S1C). Although we could not detect the distinct regulation of C/EBPβ and C/EBPδ expressions in the limb chondrocytes before and after birth, their actions on chondrocyte hypertrophy might be compensatory, especially postnatally. We are now investigating the role of C/EBPδ in the skeletal growth using the knockout mice as well as the double knockout mice of C/EBPβ and C/EBPδ.

The runt family transcription factor member Runx2 [1], [32], [33], parathyroid hormone/parathyroid hormone-related protein (PTH/PTHrP) [1], [34], and cyclic GMP-dependent protein kinase II (cGKII) [35], [36] are known as representative regulators of chondrocyte hypertrophy, and interestingly, C/EBPβ has been reported to be associated with these representative regulators. C/EBPβ acts as a co-activator of Runx2 [6], [37]. Generally, the complex of the members of the C/EBP and Runx families is known to interact in the activation of lineage-specific promoters during differentiation of osteoblasts, adipocytes, and granulocytes [6]. Unlike Runx2−/− mice that exhibit a complete lack of bone [32], C/EBPβ−/− mice showed almost normal bone, raising the possibility of functional redundancy with other isoforms such as C/EBPα or C/EBPδ in osteoblast differentiation. Contrarily, both Runx2−/− and C/EBPβ−/− mice showed impairment of chondrocyte hypertrophy during cartilage development and growth [32], [33], implicating a specific interaction between C/EBPβ and Runx2 in cartilage. In the present study, the site-directed mutagenesis in the C/EBP motif of the p57 promoter caused significant but incomplete suppression of the promoter activity induced by the C/EBPβ overexpression (Figure 5C). Actually, there is a putative Runx motif which lies close to this C/EBP motif in this region. C/EBPβ might therefore stimulate the promoter activity at the Runx motif as a co-activator of Runx2, even after the innate binding was blocked, although our luciferase assay and EMSA so far have failed to find evidence of this.

Contrarily to Runx2, PTH/PTHrP keeps chondrocytes proliferating and inhibits their hypertrophic differentiation [1], [34]. The PTH/PTHrP action via the adenyl cyclase signal in chondrocytes is reported to be dependent on the suppression of p57 expression [38], implicating a possible mediation of C/EBPβ in this pathway. However, our present study showed that neither PTH nor the adenyl cyclase activator forskolin affected the C/EBPβ protein level in cultured ATDC5 cells or the activity of the p57 promoter (−150 to +226 bp) with or without induction by C/EBPβ (Figure S2). Although C/EBPβ is therefore unlikely to mediate the p57 suppression by PTH/PTHrP directly, its possible involvement as a co-activator of Runx2 again cannot be denied here, since the PTH/PTHrP action is also at least partly dependent on the Runx2 suppression in chondrocytes [39].

cGKII is a serine/threonine kinase lying downstream of the C-type natriuretic peptide pathway which is essential for skeletal growth [40]. We and others have reported that the deficiency of cGKII in mice and rats caused dwarfism due to impaired hypertrophic differentiation of chondrocytes [35], [41], similarly to the present C/EBPβ−/− mice. Interestingly, a previous study showed that cGKII activated C/EBPβ through phosphorylation of glycogen synthase kinase-3β (GSK-3β) in osteosarcoma cells [42], and our recent study showed that cGKII induced chondrocyte hypertrophic differentiation through the GSK-3β phosphorylation [36]. These suggest a possible mediation of the present C/EBPβ-p57 signal in the cGKII-GSK-3β action. However, neither cGKII nor GSK-3β overexpression altered at least the activity of the p57 promoter (−150 to +226 bp) with or without induction by C/EBPβ (Figure S3). Moreover, there is a marked difference in the limb cartilage phenotype between cGKII−/− and C/EBPβ−/− mice. Unlike the cGKII−/− cartilage characterized by appearance of a wide abnormal intermediate layer between the proliferative and hypertrophic zones [35], [41], the C/EBPβ−/− cartilage only exhibited an elongated proliferative zone and delayed chondrocyte hypertrophy (Figure 2D). Hence, the cell cycle arrest in chondrocytes caused by C/EBPβ appears to immediately link to the start of the differentiation. The discrepancy may be due to the diversity of signaling pathway(s) lying downstream of cGKII and upstream of C/EBPβ. We previously reported that cGKII phosphorylated Sox9, an inhibitor of chondrocyte hypertrophy, and suppressed its nuclear entry [35]. Besides Sox9 and GSK-3β, vasodilator-stimulated phosphoprotein and cysteine- and glycine-rich protein 2 are putative phosphorylation targets of cGKII in other types of cells [43]. In addition to the abovementioned GSK-3β, C/EBPβ is also targeted by multiple protein kinases including protein kinase A, calmodulin-dependent protein kinase, Erk-1/2, ribosomal protein S6 kinase, and CDK2 [44].

The skeletal abnormalities of C/EBPβ−/− mice were much milder than those of the p57−/− mice which were perinatally lethal due to various defects analogous to Beckwith-Weidemann syndrome in children, including cleft palate and body wall dysplasia besides severe dwarfism [38], [45]–[47]. This may be because p57 is more crucial for chondrocyte hypertrophic differentiation than C/EBPβ whose function could be substituted by several upstream signals of p57. In fact, the C/EBPβ deficiency did not abrogate, but partially suppressed the p57 expression in chondrocytes (Figure 4B, C), while the knockdown of p57 strongly suppressed the C/EBPβ-induced hypertrophic differentiation of chondrocytes (Figure 5F). Among other cell cycle factors, mice lacking the Rb-related pocket proteins p107 and p130 show skeletal phenotype very similar to that of the p57−/− mice [48], indicating that these proteins are likely to be major downstream targets of the cyclin-CDK complexes that are inhibited by p57 in chondrocytes. More interestingly, the Cip/Kip family proteins have recently been reported to regulate pathways distinct from that of cell cycle control [14]. Since p57 supports skeletal myoblast differentiation by inhibiting phosphorylation of the key transcription factor MyoD [49], this factor might also induce chondrocyte hypertrophic differentiation by regulating crucial transcription factors like Runx2 or Sox9. This may also explain the direct linkage from the cell cycle arrest to cell differentiation by the C/EBPβ-p57 signaling, unlike the cGKII signal, as mentioned above.

We conclude that C/EBPβ directly transactivates p57 to promote transition from proliferation to hypertrophic differentiation of chondrocytes during endochondral ossification. Besides the anabolic function for physiological skeletal growth, the C/EBPβ haploinsufficiency in adult mice caused resistance to cartilage destruction during osteoarthritis progression in knee joints (Figure 6). Furthermore, C/EBPβ has been reported to be induced by proinflammatory cytokines interleukin-1 and tumor necrosis factor-α, and to mediate the decrease of articular cartilage matrix by suppressing the promoter activity of cartilage characteristic genes like cartilage-derived retinoic acid-sensitive protein and type II collagen [50]–[52]. The cytokine-induced C/EBPβ also enhances the promoter activity of prostaglandin synthetic enzymes like cyclooxygenase-2 and phospholipase A2 [53], [54] and proteinases like aggrecanase-1 and matrix metalloproteinase-1 [55], [56] in chondrocytes. These lines of evidence indicate that the C/EBPβ-p57 signal could be a therapeutic target of inflammatory and degenerative joint disorders as well as skeletal growth retardation.

## Materials and Methods

### Ethics statement

All experiments were performed according to the protocol approved by the Animal Care and Use Committee of the University of Tokyo.

### Animals

C/EBPβ deficient mice, kindly provided by Dr. Shizuo Akira (University of Osaka), were maintained in a C57BL/6 background. In each experiment, we compared C/EBPβ−/− or C/EBPβ+/− mice with the wild-type littermates.

### Histological analysis

The whole skeletons of WT and C/EBPβ−/− littermate embryos (E16.5) were fixed in 99.5% ethanol, transferred to acetone, and stained in a solution containing Alizarin red S and Alcian blue 8GX (Sigma). For histological analysis, tibial limbs were fixed in 4% paraformaldehyde (PFA) buffered with PBS and sectioned in 5-µm slices. Hematoxylin-eosin (HE) stainings were performed according to standard protocols. Alcian blue/von Kossa double stainings were performed with 1% Alcian blue 8GX in 3% acetate and with 5% silver nitrate. For immunohistochemistry, the sections were incubated with antibodies to C/EBPβ (C-19), p57 (C-20), Ihh (C-15), and C/EBPδ (M-17) (Santa Cruz Biotechnology Inc.) diluted 1∶500 in blocking reagent. The localization of C/EBPβ was detected with HRP-conjugated secondary antibody (Promega). For fluorescent visualization, a secondary antibody conjugated with Alexa Fluor 488 (Invitrogen) was used. The p57 detection was performed using a CSA II, Biotin-Free Catalyzed Amplification System (DAKO). In situ hybridization was performed, as we reported previously [21]. Briefly, hybridization with complementary digoxigenin (DIG)-labeled for mouse type X collagen was performed in a humidified chamber for 16 h at 52°C. For the detection of DIG-labeled probes, slides were incubated with HRP-conjugated anti-DIG rabbit polyclonal antibody (Dakopatts). The sections were immersed in a diaminobenzidine solution to visualize immunoreactivity. For BrdU labeling, we injected BrdU (Sigma) intraperitoneally to pregnant mice prior to sacrifice, and the sections were stained using a BrdU Immunohistochemistry System (Calbiochem) and Alexa Fluor 568 (Molecular Probes).

### Cell cultures

Primary chondrocytes were isolated from the ribs of mouse embryos as previously described [57]. The primary chondrocytes, HuH-7 cells and C3H10T1/2 cells were cultured in DMEM with 10% FBS. ATDC5 cells were maintained in DMEM/F12 with 5% FBS. To induce hypertrophic differentiation, the ATDC5 cells were cultured for 3 weeks with insulin.

### Plasmids and viral vectors

C/EBPβ and p57 cDNA were cloned into pMx vectors, and retroviral vectors were generated using plat-E cells [58]. The siRNA sequence was designed for the mouse p57 gene (NM_009876.3: nucleotides 925–946 and 307–328) and GFP as previously described [59] and ligated into piGENEmU6 vector (iGENE Therapeutics). The siRNA sequence combined with the promoter was then inserted into a retroviral pMx vector. The adenovirus C/EBPβ and LacZ expression vector were synthesized using an Adeno-X expression system (Clontech). Two weeks after transfection, the cells were harvested and used for subsequent assays. cDNA of cGKII and GSK-3β was ligated into pCMV-HA (Invitrogen).

### Cell proliferation assay

Primary chondrocytes were inoculated at 10^3^ cells per well in a 96-well plate. The proliferation of cells was examined, using an XTT Assay Kit (Roche) at the indicated time point. The absorbance of the product was quantified using a MTP-300 microplate reader (Corona Electric). For BrdU detection analysis, we labeled the chondrocytes with 10 µM BrdU (Sigma) for 18 h and the cells were stained using a BrdU Immunohistochemistry System (Calbiochem).

### Chondrocyte differentiation assay

Primary chondrocytes were cultured for two weeks after confluency, and the total RNA was extracted to assess the COL10, MMP13, and VEGF mRNA levels. For the ALP staining, cells were stained with a solution containing 0.01% Naphthol AS-MX phosphate disodium salt (Sigma), 1% N, N-dimethyl-formamide (Wako), and 0.06% fast blue BB (Sigma). For the Alizarin red S staining, cells were stained with 2% Alizarin red S solution (Sigma).

### Flow cytometric analysis

C3H10T1/2 cells with retroviral transfection with C/EBPβ or GFP were incubated for 18 h in the presence of 0.2 µM nocodazole for synchronization at the G2/M phase. Then, cells were suspended in citrate buffer and stained with propidium iodide. DNA content was analyzed with EPICS XL and XL EXPO32 instruments (Beckman).

### Real-time RT-PCR

The total RNA was extracted using an ISOGEN Kit (Wako) and an RNeasy Mini Kit (QIAGEN). One µg of RNA was reverse-transcribed with a Takara RNA PCR Kit (AMV) ver.2.1 (Takara) to generate single-stranded cDNA. PCR was performed with an ABI Prism 7000 Sequence Detection System (Applied Biosystems). All reactions were run in triplicate. Primer sequence information is available upon request.

### Luciferase assay

The human p57 promoter regions were cloned into the pGL4.10 vector (Promega). Other deletion constructs were created by the PCR technique. Tandem-repeat constructs were created by ligating the double strand oligonucleotides from −150 to −130 bp into pGL4.10 vector. Transfection in HuH-7 and ATDC5 cells was performed in quadruplicate using Fugene (Roche). For PTH or forskolin stimulation, cells were cultured with PTH (10 nM) or forskolin (10 nM) at the time of transfection. The luciferase assay was performed with a PicaGene Dual SeaPansy Luminescence Kit (Toyo Ink) and GloMax™ 96 Microplate Luminometer (Promega).

### Electrophoretic Mobility Shift Assay (EMSA)

Nuclear extracts were prepared from ATDC5 cells adenovirally transfected with C/EBPβ. Oligonucleotide probes of the −160 to −120 bp region sequence in the p57 promoter were labeled with digoxigenin by using a DIG gel shift kit (Roche). For competition analyses, 50-fold excess of unlabeled competitor probe was included in the binding reaction. For the supershift experiments, 1 µL of an antibody to C/EBPβ (Santa Cruz Biotechnology Inc.) was added.

### Microarray analysis

Total RNA was isolated from C3H10T1/2 cells with retroviral introduction of C/EBPβ or the empty vector after 1 week of culture. The microarray experiment was performed using the Gene Chip Mouse Genome 430 2.0 Array (Affymetrix), scanned by GeneChip Scanner 3000, and analyzed using GCOS ver 1.4 software.

### Immunoblotting

ATDC5 cells were cultured with PTH (10 nM) or forskolin (10 nM) for 0 to 30 min, and then their cytoplasmic and nuclear proteins were extracted with an NE-PER (Pierce Chemical). For immunoblot analysis, lysates were fractionated by SDS-PAGE and transferred onto nitrocellulose membranes (BIO-RAD). The membranes were incubated with an antibody to C/EBPβ (Santa Cruz), or an antibody to actin (Sigma). Immunoreactive bands were visualized with ECL Plus (Amersham Biosciences).

### Osteoarthritis experiment

The surgical procedure to create an osteoarthritis experimental model was performed on 8-week-old male mice, as we have reported previously [17], [20], [21]. At the indicated time points after surgery, the mice were killed, and the entire knee joints were dissected and fixed for 24 h at 4°C in 4% PFA. The specimens were decalcified for 2 weeks with 10% EDTA (pH 7.4) at 4°C and sectioned in 5-µm slices. Sections were stained with Safranin O–fast green. Destruction of cartilage was quantified according to the OARSI grading system [22]. For immunohistochemistry, the sections were incubated with antibodies to C/EBPβ (Santa Cruz), COL10 (LSL) or the nonimmune rabbit IgG as the negative control diluted 1∶500 in blocking reagent, and the localization was detected with HRP-conjugated secondary antibody (Promega).

### Statistical analysis

Means of groups were compared by ANOVA, and significance of differences was determined by post-hoc testing using Bonferroni's method.

## Supporting Information

Table S1 Microarray analysis shows the changes in expression of cell cycle factors by C/EBPβ overexpression. Ratios of mRNA levels in C3H10T1/2 cells with retroviral introduction of C/EBPβ in comparison with the control empty vector were determined by Gene Chip Mouse Genome 430 2.0 Array (Affymetrix). All results of the microarray analysis are provided at ArrayExpress (accession number: E-MEXP-1984).
(0.02 MB PDF)

Figure S1 C/EBPδ shows similar expression and function in chondrocytes to those of C/EBPβ. (A) Immunostaining with an antibody to C/EBPδ in the tibial cartilage of wild-type (WT) and C/EBPβ−/− littermates (E16.5). Red, blue, and green bars indicate layers of proliferative zone, hypertrophic zone, and bone area, respectively. Scale bars, 100 mm. (B) Relative mRNA levels of COL10, MMP13, and VEGF of ATDC5 cells with retroviral transfection of C/EBPδ or the control GFP determined by real-time RT-PCR at 2 weeks of culture after confluency. (C) The p57 promoter activity in ATDC5 cells transfected with luciferase-reporter construct containing the 5′-flanking sequences from −150 to +226 bp of the p57 promoter with effector plasmid expressing C/EBPδ, C/EBPβ, or the control GFP. All data are expressed as means (symbols or bars)±SEM (error bars) of 6 wells per group. *P<0.01 vs. GFP.
(0.68 MB PDF)

Figure S2 PTH and forskolin have no effects on C/EBPβ protein level and p57 promoter activity. (A) Time course of C/EBPβ protein level in cultured ATDC5 cells. After the indicated time of treatment with PTH (10 nM) and forskolin (10 nM), the C/EBPβ protein levels in the cytoplasmic fraction (C) and nuclear fraction (N) were determined by immunoblotting with an antibody to C/EBPβ or actin as the loading control. (B) Effects of PTH, forskolin or the control on HuH-7 cells transfected with luciferase-reporter construct containing the 5′-flanking fragment (−150 to +226 bp) of the p57 promoter with effector plasmid expressing C/EBPβ or GFP as the control. The promoter activity was determined by the luciferase assay after 2 d of treatment.
(0.07 MB PDF)

Figure S3 Effects of cGKII and GSK-3β overexpression on p57 promoter activity. The promoter activity was determined by the luciferase assay in HuH-7 cells transfected with luciferase-reporter construct containing the 5′-flanking fragment (−150 to +226 bp) of the p57 promoter with effector plasmid expressing C/EBPβ or GFP as the control.
(0.02 MB PDF)
