# Supplementary material for: C/EBPβ Promotes Transition from Proliferation to Hypertrophic Differentiation of Chondrocytes through Transactivation of p57Kip2
Source: PLoS One. 2009 Feb 20;4(2):e4543. doi: 10.1371/journal.pone.0004543 (PMC2638010; doi:10.1371/journal.pone.0004543)
Supplement: Table S1 — Microarray analysis shows the changes in expression of cell cycle factors by C/EBPβ overexpression. Ratios of mRNA levels in C3H10T1/2 cells with retroviral introduction of C/EBPβ in comparison with the control empty vector were determined by Gene Chip Mouse Genome 430 2.0 Array (Affymetrix). All results of the microarray analysis are provided at ArrayExpress (accession number: E-MEXP-1984). (0.02 MB PDF) [file pone.0004543.s001.pdf]

Table S1. Microarray analysis shows the changes in expression of cell cycle factors by C/EBPβ overexpression

Gene title	GenBank™ accession no.	Ratio (C/EBPβ v.s. Mock)
**Cyclin**		
cyclin A1	NM_007628.1	1.149
cyclin A2	NM_009828.1	0.574
cyclin B1	AU015121	0.467
cyclin B2	AK013312.1	0.500
cyclin D1	NM_007631.1	0.500
cyclin D2	NM_009829.1	0.933
cyclin D3	NM_007632.1	0.933
cyclin E1	NM_007633.1	0.707
cyclin E2	AF091432.1	0.660
cyclin F	NM_007634.1	0.812
cyclin G1	NM_009831.1	1.414
cyclin G2	NM_007635.1	0.933
cyclin H	NM_023243.1	1.072
cyclin I	NM_017367.1	0.933
cyclin K	BC027297.1	0.812
cyclin T1	NM_009833.1	0.933
cyclin T2	AK013634.1	1.000
**Cyclin-dependent kinase (Cdk)**		
Cdk 2	NM_016756.1	1.072
Cdk 4	NM_009870.1	0.758
Cdk 5	NM_007668.1	1.149
Cdk 6	NM_009873.1	1.072
**Cyclin-dependent kinase inhibitor**		
**Cip/Kip**		
Cdkn 1A (p21)	NM_007669.1	1.625
Cdkn 1B (p27)	NM_009875.1	0.707
Cdkn 1C (p57)	NM_009876.1	1.866
**INK4**		
Cdkn 2A (p16)	NM_009877.1	2.144
Cdkn 2B (p15)	AF059567.1	0.616
Cdkn 2C (p18)	AI323293	0.707
Cdkn 2D (p19)	BC013898.1	0.758

Ratios of mRNA levels in C3H10T1/2 cells with retroviral introduction of C/EBPβ in comparison with the control empty vector were determined by Gene Chip Mouse Genome 430 2.0 Array (Affymetrix). All results of the microarray analysis are provided at ArrayExpress (accession number: E-MEXP-1984).
