# Supplementary figures and images for: C/EBPβ Promotes Transition from Proliferation to Hypertrophic Differentiation of Chondrocytes through Transactivation of p57Kip2
Source: PLoS One. 2009 Feb 20;4(2):e4543. doi: 10.1371/journal.pone.0004543 (PMC2638010; doi:10.1371/journal.pone.0004543)

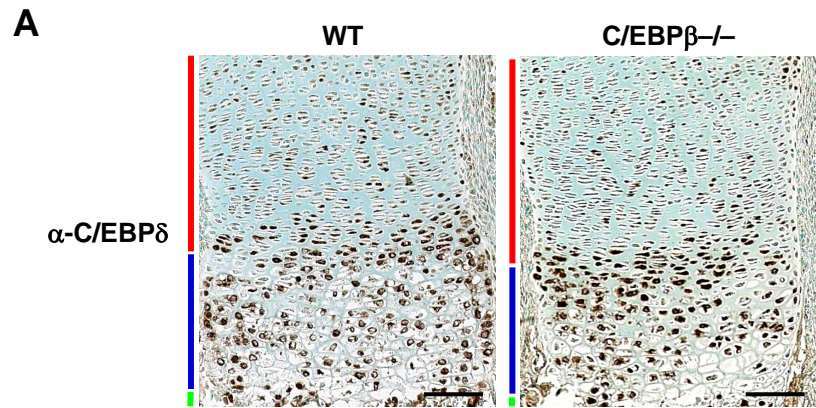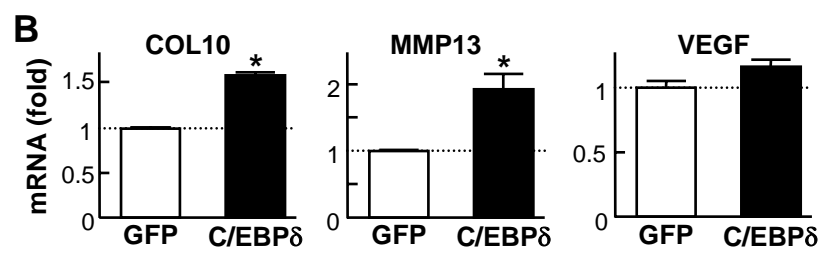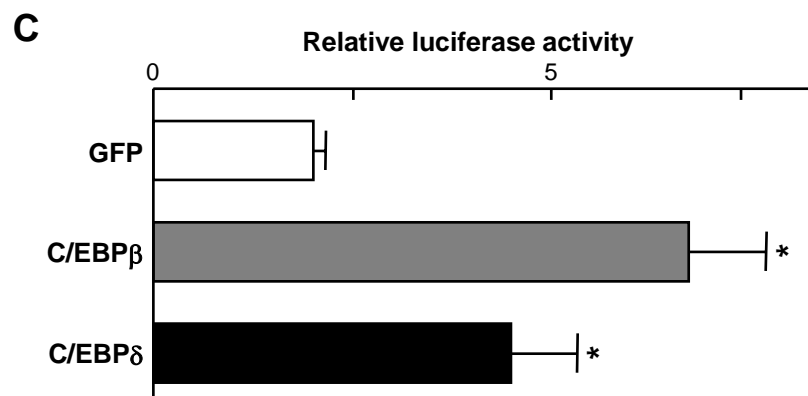

Supplement: Figure S1 — C/EBPδ shows similar expression and function in chondrocytes to those of C/EBPβ. (A) Immunostaining with an antibody to C/EBPδ in the tibial cartilage of wild-type (WT) and C/EBPβ−/− littermates (E16.5). Red, blue, and green bars indicate layers of proliferative zone, hypertrophic zone, and bone area, respectively. Scale bars, 100 mm. (B) Relative mRNA levels of COL10, MMP13, and VEGF of ATDC5 cells with retroviral transfection of C/EBPδ or the control GFP determined by real-time RT-PCR at 2 weeks of culture after confluency. (C) The p57 promoter activity in ATDC5 cells transfected with luciferase-reporter construct containing the 5′-flanking sequences from −150 to +226 bp of the p57 promoter with effector plasmid expressing C/EBPδ, C/EBPβ, or the control GFP. All data are expressed as means (symbols or bars)±SEM (error bars) of 6 wells per group. *P<0.01 vs. GFP. (0.68 MB PDF) [file pone.0004543.s002.pdf]

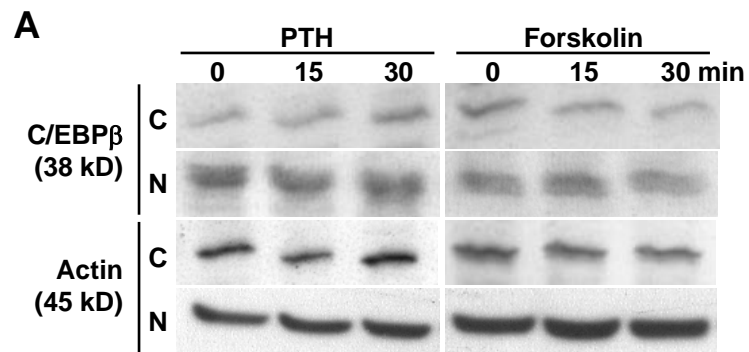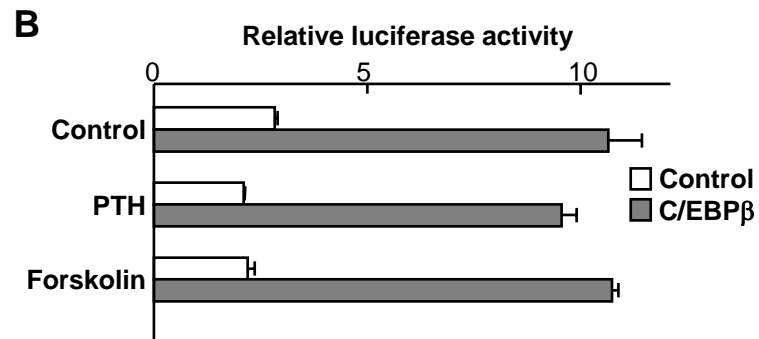

Supplement: Figure S2 — PTH and forskolin have no effects on C/EBPβ protein level and p57 promoter activity. (A) Time course of C/EBPβ protein level in cultured ATDC5 cells. After the indicated time of treatment with PTH (10 nM) and forskolin (10 nM), the C/EBPβ protein levels in the cytoplasmic fraction (C) and nuclear fraction (N) were determined by immunoblotting with an antibody to C/EBPβ or actin as the loading control. (B) Effects of PTH, forskolin or the control on HuH-7 cells transfected with luciferase-reporter construct containing the 5′-flanking fragment (−150 to +226 bp) of the p57 promoter with effector plasmid expressing C/EBPβ or GFP as the control. The promoter activity was determined by the luciferase assay after 2 d of treatment. (0.07 MB PDF) [file pone.0004543.s003.pdf]

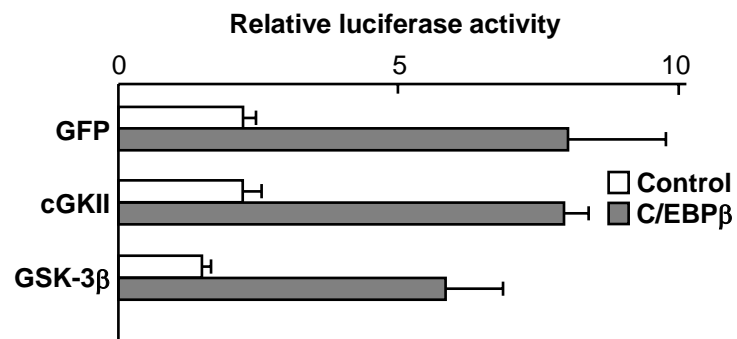

Supplement: Figure S3 — Effects of cGKII and GSK-3β overexpression on p57 promoter activity. The promoter activity was determined by the luciferase assay in HuH-7 cells transfected with luciferase-reporter construct containing the 5′-flanking fragment (−150 to +226 bp) of the p57 promoter with effector plasmid expressing C/EBPβ or GFP as the control. (0.02 MB PDF) [file pone.0004543.s004.pdf]
